# The therapeutic and prognostic implications of immunobiology in colorectal cancer: a review

**DOI:** 10.1038/s41416-021-01475-x

**Published:** 2021-07-23

**Authors:** Alexandra M. Zaborowski, Des C. Winter, Lydia Lynch

**Affiliations:** 1 Centre for Colorectal Disease, St. Vincent’s University Hospital, Dublin 4, Ireland; 2 School of Biochemistry and Immunology, Trinity College Dublin, Dublin, Ireland; 3 School of Medicine, University College Dublin, Dublin 4, Ireland; 4 Harvard Institutes of Medicine, Harvard Medical School, Boston, MA USA

**Keywords:** Colorectal cancer, Tumour immunology

## Abstract

Colorectal cancer represents the second leading cause of cancer-related death worldwide. The therapeutic field of immuno-oncology has rapidly gained momentum, with strikingly promising results observed in clinical practice. Increasing emphasis has been placed on the role of the immune response in tumorigenesis, therapy and predicting prognosis. Enhanced understanding of the dynamic and complex tumour-immune microenvironment has enabled the development of molecularly directed, individualised treatment. Analysis of intra-tumoural lymphocyte infiltration and the dichotomisation of colorectal cancer into microsatellite stable and unstable disease has important therapeutic and prognostic implications, with potential to capitalise further on this data. This review discusses the latest evidence surrounding the tumour biology and immune landscape of colorectal cancer, novel immunotherapies and the interaction of the immune system with each apex of the tripartite of cancer management (oncotherapeutics, radiotherapy and surgery). By utilising the synergy of chemotherapeutic agents and immunotherapies, and identifying prognostic and predictive immunological biomarkers, we may enter an era of unprecedented disease control, survivorship and cure rates.

## Introduction

Colorectal cancer (CRC) represents the most common intra-abdominal malignancy and second leading cause of cancer-related death worldwide [1]. Although complete surgical excision is central to curative treatment, improved oncological outcomes have been achieved by the addition of systemic neoadjuvant and adjuvant therapy [2–5]. In spite of these advances, distant disease failure rates are disappointingly high, ranging between 20 and 30% in high income countries where multimodal approaches are possible, and account for most cancer-specific mortality [2]. Enhanced understanding of the dynamic and complex tumour microenvironment enabled development of molecularly directed, individualised treatment. Increasing emphasis has been placed on the role of the immune response in tumorigenesis, therapy, and predicting prognosis. Modifying anti-tumour immunity provides a promising oncotherapeutic strategy with some strikingly encouraging results observed in a range of malignancies including CRC.

## Cancer and the immune response

The ability of the immune system to paradoxically constrain and promote tumour development and progression was first described by Sir Frank MacFarlane Burnet in 1957 [6]. This process, referred to as cancer immunoediting, consists of 3 phases: elimination, equilibrium and escape [7]. During the elimination phase the innate and adaptive immune systems work synergistically to recognise and destroy tumour cells. In the equilibrium phase, tumour cells that survived elimination coexist with the immune system and are kept functionally dormant by a balance of anti-tumour and pro-tumour cytokines. Over time, continuous immune pressure results in genetic and epigenetic changes within the tumour cells that lead to the emergence of immune-resistant variants and progression to the escape phase. The escape phase, recognised as one of the ‘Hallmarks of Cancer’ [8], is characterised by tumour cell proliferation and metastasis. Tumour cells evade immune recognition through loss of antigen presentation and create an immunosuppressive microenvironment via several mechanisms, including production of cytokines TGFß and IL-10, and deactivation of the cytotoxic T-cell response [9]. The development of single cell genomics has since enabled the evaluation of immune cells in the tumour microenvironment and their evolution over time.

Even with the advanced technology of the modern subcellular era, the immune microenvironment of cancer is multi-layered, complex, and incompletely understood [10]. Just as each organ is immunologically distinct, so too are the tumours that develop in them. The diversity of tumoural immune infiltration has led increasingly to the nomenclature ‘*hot*’ or ‘*cold*’ tumours, where ‘*hot*’ tumours are those characterised by high immune infiltrate, particularly cytotoxic T-cell infiltration and ‘*cold*’ tumours demonstrate an absence of or poor T-cell infiltration [11]. The Immunoscore, a simple scoring system developed to measure intra-tumoural immune response, is based on the quantification of cytotoxic and memory T cells in the core of the tumour and its invasive margin [12]. It provides a score ranging from 0 (low densities of both lymphocyte populations in both regions) to 4 (high densities in both regions). Importantly, the Immunoscore has been internationally validated as an independent predictor of disease-specific survival in colorectal cancer (CRC), superior to the classical TNM staging system [13].

Developments in digital computational pathology now also allow comprehensive analysis of the spatial organisation of immune cells in the tumour microenvironment. Using data from The Cancer Genome Atlas (TCGA), computational stains were analysed to characterise TIL patterns for almost 5000 patients with 13 cancer types [14]. TIL patterns were assigned one of five categories on the basis of strength of immune response, qualitative pattern of immune response (localised, diffuse), and proportion of tumour composed of lymphocytes. Among the tumour types analysed was rectal cancer which was most commonly within the category ‘brisk, diffuse’; characterised by a moderate to strong immune response, diffuse and dense infiltrate, and >30% TILs in the intra-tumoural component. Spatial patterns may be associated with response to therapy and survival, potentially providing valuable therapeutic and prognostic information. Furthermore, development of image-based molecular profiling using standard histology sections and deep learning may facilitate widespread implementation of precision therapy on the basis of gene expression [15].

The cellular landscape of CRC has been further defined by the identification of specific immune signatures. IFN-γ dominant profiles are associated with improved survival whilst IL-17 dominant patterns may signify a poor prognosis [16]. CRC is associated with a wound-healing immune phenotype characterised by a high proliferative activity and intra-tumoural heterogeneity with a low T helper type 1 (pro-inflammatory) to T helper type 2 (anti-inflammatory) ratio [17]. Given the function of the lower gastrointestinal tract, repeated exposure to pathogens, and the constant immune-commensal homeostatic communication, tissue-resident immune cells are tightly regulated to ensure homeostasis. This basal tuning of the immune system with capacity to mount appropriate and regulated responses should be considered when designing immune-modifying therapies for CRC. Modifying tissue-resident immunity may disrupt homeostasis leading to autoimmunity and significant side effects.

## Tumour biology of colorectal cancer

Intra-tumoural immune responses are influenced by a tumour biology that displays considerable heterogeneity as it develops via several distinct oncogenic pathways [18]. The majority of CRCs (80–85%) are derived by chromosomal instability (CIN), and have been further subdivided into three consensus molecular subtypes on the basis of distinguishing molecular characteristics: [1] CMS2 ‘*canonical*’ with WNT and MYC activation [2]; CMS3 ‘*metabolic*’ with metabolic dysregulation and KRAS mutations and [3] CMS4 ‘*mesenchymal*’ with marked stromal infiltration, TGFß activation and angiogenesis [19]. The remaining 15–20% (CMS1) develop via defective DNA mismatch repair (MMR) leading to microsatellite instability (MSI). MSI may be caused by sporadic events (e.g. epigenetic silencing of the MLH1 gene) or by constitutive mutations in one of the MMR genes—the most common of which are MLH1, MSH2, MSH6 and PMS2—resulting in the most common hereditary cancer syndrome (Lynch Syndrome) [20]. Inability to correct mismatches in DNA replication leads to the accumulation of multiple insertion and deletion mutations at coding microsatellites which in turn generates highly immunogenic frameshift peptide (FSP) neoantigens [21, 22]. It is thought that this high load of neoantigens provokes the strong local immune response observed in MSI tumours. This robust peritumoural immune reaction is characterised by dense cytotoxic T-cell infiltration associated with a Crohn’s-like lymphocytic reaction and a favourable pro-inflammatory, IFNγ dominant Th1 response [23]. Analysis of immune landscape on the basis on CMS, revealed enrichment of CD8+ T cells, natural killer cells and γδT cells, and upregulation of checkpoint regulators Lag3 and TIGIT in CMS1 tumours [24].

The enhanced immunogenicity of MSI tumours may in part explain their favourable prognosis compared to those that are microsatellite stable (MSS). MSI tumours are less likely to metastasise to lymph nodes and distant organs, and are associated with better stage-specific survival [25]. Interestingly, a single centre study reported the ‘protective’ effect of MSI may be lost in node positive disease with disease-specific survival comparable to that of MSS tumours of the same stage [26]. MSI status may also play an important role in therapeutic decision making. The predictive role of MSI for response to fluoropyrimidine-based chemotherapy is unclear with conflicting data [27–29]. A meta-analysis of 7 studies including 3690 patients found similar disease-free survival in treated and untreated patients with MSI-High (MSI-H) tumours [30] (Table 1). Despite a strong tumour-immune infiltrate, the immune system does not appear to eradicate the disease, suggesting MSI tumours may acquire mechanisms to defy immunological control.

**Table 1:** Comparison of clinical, histopathological and immunological features of colorectal tumours with microsatellite stability (MSS) and instability (MSI).

Clinical feature	MSS	MSI
Localisation	Distal colon and rectum	Proximal colon
Histopathology	Mostly glandular, Well to moderately differentiated	Poorly differentiated, Mucinous, signet ring, medullary
Distant metastasis potential	High	Low
Response to 5-FU-based chemotherapy	Good	Poor
Response to inhibitory checkpoint therapy	Poor	Good
Lymphocytic infiltration	Low-moderate	High
Overall mutation rate	Low	Very high

Several mechanisms of MSI-mediated immune evasion have been described including alterations in tumour-specific antigen presentation. One of the most common is loss of HLA class I antigen due to mutations in the beta-2-microglobulin gene (B2M), occurring in 30% of MSI tumours and rarely in MSS [31]. Such mutations are associated with reduced risk of local and distant disease recurrence [32, 33]. The majority of cases studied were pathological stage II and whether this protective effect extends to stage III (node positive) disease is unclear, as is the exact cellular mechanism of protection. Whilst absence of HLA class 1 expression enables tumour cells to avoid recognition by cytotoxic T cells, natural killer cells (NK) become activated [34]. This important surveillance system termed ‘missing self’ results in NK-mediated destruction of circulating tumour cells and may account for the reduced incidence of disease recurrence in patients with mutations in B2M [35]. These data suggest B2M testing may represent a useful prognostic tool in MSI tumours.

The immune landscape of Lynch Syndrome-associated CRC (i.e. cancer arising in the context of LS) and sporadic MSI CRC differ, possibly due to differences in molecular pathogenesis. The lifetime risk of developing CRC in LS is between 50–70%, despite what might be expected to be inevitable, suggesting that immune surveillance may protect against tumour development [36]. Density of T-cell infiltration appears to be higher in LS-associated MSI CRC compared with sporadic MSI CRC [37–39]. The presence of frameshift neoantigen-specific T-cell reactivity in healthy patients with LS suggests pre-sensitisation of the immune system and may account for the more intense intra-tumoural T-cell response observed in LS-associated CRC [40]. Investigation into whether vaccination with MMR deficiency-induced neoantigens could ignite an adaptive immune response preventing tumorigenesis is ongoing, and promising results have been demonstrated in a small clinical trial without occurrence of serious adverse events [41].

## Therapeutic implications

The discovery of the immune checkpoints, Programmed cell death protein (PD-1) and Cytotoxic T-lymphocyte-associated protein 4 (CTLA-4) and their role in cancer immunology represent promising developments in oncotherapeutics, recognised by the Nobel Prize in 2018. PD-1 is one of a number of inhibitory checkpoints which are proteins expressed on the surface of activated T cells following T-cell receptor (TCR) engagement with tumour antigens. Binding of the checkpoints and their ligands (e.g. PD-L1 or PD-L2) suppresses T-cell effector function. Tumour cells (and other pro-tumour-immune cells) can limit anti-tumour response via upregulation of these ligands [42]. Chronic antigen exposure and TCR signalling leads to persistent checkpoint expression on tumour-infiltrating lymphocytes (TILs) which evokes an ‘exhausted’ or dysfunctional state, characterised by reduced proliferative capacity, cytokine production or cytotoxicity [43]. T-cell exhaustion represents a distinct and stable state of differentiation [44]. Phenotypic and functional heterogeneity exists among exhausted TILs giving rise to two subpopulations [45]. ‘Progenitor’ T cells express intermediate levels of PD-1, whilst those that are terminally exhausted exhibit high levels of PD-1 and other co-inhibitory receptors (T-cell immunoglobulin (Ig) mucin 3 (TIM-3), lymphocyte-activation gene 3 (LAG-3) and T-cell immunoreceptor with Ig and ITIM domains (TIGIT)) [46]. Monoclonal antibodies against checkpoint receptors can block this interaction to reinvigorate effector function and enhance anti-tumour responses. Recent data suggests that infiltrating PD-1 + T cells rather than tumour resident T cells are reinvigorated [47].

**Fig. 1:**
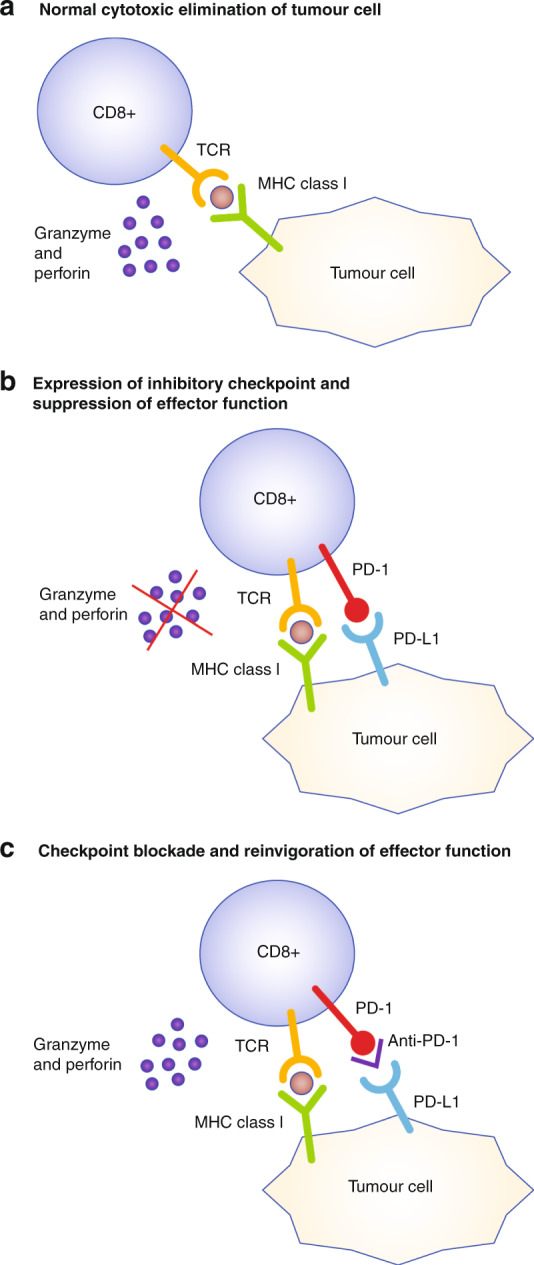
Activation of CD8+ T cells in the tumour microenvironment, and mechanisms of inhibitory checkpoint blockade. **a** Normal cytotoxic elimination of tumour cell. **b** Expression of inhibitory checkpoint and suppression of T-cell effector function. **c** Checkpoint blockade and reinvigoration of effector function.

Several biomarkers to identify TILs responsive to checkpoint therapy have shown promising results in pre-clinical studies. For example, the expression of chemokine receptor CXCR5 and transcription factor Tcf-1 appear to identify the progenitor subpopulation of CD8 + TILs that demonstrate increased effector function upon checkpoint blockade [45, 48] (Fig. 1). Additional biomarkers are rapidly emerging from sequencing of responders and non-responders.

The immunobiology of CRC has important therapeutic implications as microsatellite status appears to predict response to immunotherapy with checkpoint blockade. Clinical efficacy is predominantly limited to MSI tumours whilst MSS tumours are largely refractory [49]. As pre-existing T-cell infiltration is a prerequisite for checkpoint therapy to be effective, MSI tumours represent ideal targets [50]. Furthermore, they exhibit higher levels of inhibitory checkpoint expression compared to MSS [23]. Yet in spite of the marked local immune reaction, clinical efficacy varies. Two phase II pembrolizumab trials (KEYNOTE-016 and KEYNOTE-164) and one phase II nivolumab trial (CheckMate-142) evaluated checkpoint therapy in patients with previously treated MSI metastatic CRC [51–53]. Improved progression free survival (PFS) observed in these trials led to FDA approval in select patients. The recent KEYNOTE-177 trial reported superior PFS with pembrolizumab compared to chemotherapy ± bevacizumab or cetuximab (median 16.5 vs 8.2 months; HR 0.60; 95% CI 0.45–0.80, *p* = 0.0002) as first-line therapy for patients with MSI-H metastatic CRC [54] In light of these results, monotherapy with pembrolizumab has now been approved for untreated patients with MSI-H metastatic CRC.

Checkpoint therapy has also been investigated in the setting of primary CRC. In the NICHE study assessing dual agent inhibitory checkpoint blockade (PD-1 and CTLA-4), tumour regression was observed in 100% of MSI tumours, with a major pathological response (MPR ≤ 10% residual viable tumour) rate of 95% and a pathological complete response (pCR) rate of 60% [55]. In MSS tumours, 4/15 (27%) demonstrated tumour regression with 3 MPRs and 1 partial response. CD8 + PD-1 + T-cell infiltration was predictive of response in MSS tumours. It is plausible that response to checkpoint therapy is not only related to density of T-cell infiltration and T-cell activation but also the trajectory of tumour-infiltrating lymphocytes (TILs) along the spectrum of dysfunctional states.

MSS tumours represent the greatest clinical challenge in CRC as their immune microenvironment remains poorly understood. Limited response to checkpoint therapy has led to the assumption that these tumours are immunologically ‘cold’. Their low mutational burden compared to MSI tumours is thought to hinder stimulation of a local immune response allowing these tumours evade recognition by the immune system. A large international multicentre study evaluating the Immunoscore in non-metastatic colon cancer however, found that almost three in four MSS tumours were associated with an intermediate or high Immunoscore (compared to 83% of MSI tumours) [13]. A high Immunoscore was observed in 21% of MSS compared to 45% of MSI. These data suggest that a subset of immunologically *‘hot’* MSS tumours and *‘cold’* MSI tumours exist [13]. The Immunoscore was a stronger predictor of disease-specific survival than microsatellite status, which alone does not predict density of intra-tumoural T-cell infiltration. Similar findings were observed in a prospective cohort study of 1265 patients with stage II/III CRC [56]. Furthermore, pathological response to immunotherapy with checkpoint blockade has been observed in MSS tumours despite a significantly lower mutational burden than MSI tumours [55].

Although clinical efficacy with checkpoint therapy is limited in MSS tumours, it is notable that up to 1 in 4 demonstrate a response [55]. Notably, checkpoint therapy trials have not stratified patients based on pre-treatment tumour-immune infiltration or CD8 + PD-1 expression. Hence, a subset of MSS tumours may be good candidates for checkpoint blockade. Understanding the underlying mechanisms would enable more accurate selection of those likely to derive a meaningful benefit, in addition to enhancing therapeutic effect. One such potential strategy is combination therapy. Analogous to bacteria increasing their mutation rate when exposed to antibiotics, it has been postulated that tumour cells (in response to targeted therapies) may undergo a transient increase in genomic instability resulting in de novo mutagenesis [57]. Tumour cells that survive the toxic effects of targeted therapy may act as a reservoir from which genetically distinct derivatives develop. EGFR/BRAF inhibition has been shown to downregulate MMR proteins in human colorectal tumours, triggering microsatellite instability and increasing mutagenesis [57]. Once the tumour cells adapted to be able to survive in the presence of the drug, mutagenesis reverted back to baseline. EGFR/BRAF inhibition may render a tumour responsive to checkpoint blockade by temporarily converting an MSS tumour into an immunogenic MSI tumour and igniting a lymphocytic immune response. Synergism between checkpoint blockade and anti-angiogenic agents is also being investigated. The phase III COMMIT trial is currently evaluating the efficacy of atezolizumab (anti-PD-L1) and bevacizumab as first-line therapy in patients with MSI metastatic CRC [58]. Combination of therapy of targeted agents with checkpoint blockade may represent a promising strategy for MSS tumours.

## Adoptive cell therapy

Adoptive T-cell (ACT) therapy represents a cell-based strategy to modify the immune system and increase anti-tumour response via the infusion of autologous or allogenic T cells [59]. There are currently four main methods for ACT; use of tumour-infiltrating lymphocytes (TILs), insertion of chimeric antigen receptor (CAR), modification of T-cell receptors (TCR), or expansion and infusion of allogenic or autologous cytotoxic T or NK cells without known antigen specificity [60]. Although successful in haematological malignancies, clinical data in CRC specifically is sparse with the focus mainly on CAR-T-cell therapy.

In CRC, the targets of CAR-T-cell therapy include carcinoembryonic antigens (CEA), guanylyl cyclase C (GUCY2C), tumour-associated glycoprotein (TAG72) [144], epithelial cell adhesion molecule (EpCAM), NK cell surface receptor ligands (NKG2DLs) and six unique long 16 binding protein (UL-BP1-6) [60]. These molecules are normally expressed at low levels in healthy cells and upregulated in tumour cells. Although CAR-T-cell therapy has been shown to successfully induce tumour regression, a concerning side effect of this treatment is the development of severe colitis due to the presence of these targets on normal gastrointestinal mucosal cells [61, 62]. Furthermore, efficacy of CAR-T-cell therapy is reliant on the infiltration of T cells through an outer fibrous matrix into the tumour core. To date, CAR-T-cell therapy remains most efficacious in haematological malignancy with very limited success in solid tumours.

## The microbiome and the immune response

Although microsatellite status is a potential biomarker of response to immunotherapy, in isolation it does not accurately predict responders. The complex, dynamic and heterogeneous nature of the tumour-immune microenvironment and governing molecular mechanisms, suggest additional biological variables should be taken into account in patient selection.

Data from in vitro, murine and cross-sectional human studies demonstrate that the gut microbiome is involved in the aetiopathogenesis of CRC. Dynamic and evolving symbiotic relationships between key organisms can lead to the remodelling of the host microbial ecosystem towards an oncogenic phenotype [63].

There is increasing pre-clinical and clinical evidence supporting the role of the gut microbiome in modulating immune response and efficacy of immunotherapy. A mutualistic relationship between the immune system and microbiome is essential to maintain homeostasis, with the microbiome influencing innate and adaptive immunity at local and systemic level [64]. Responders to PD-1 blockade for melanoma appear to have differing gut bacterial taxa compared to non-responders, with enrichment of *Bifidobacterium longum, Collinsella aerofaciens* and *Enterococcus faecium* in stool samples [65]. Patients with a favourable gut microbiota demonstrated increased density of CD8 + cytotoxic T cells and CD4 + regulatory T cells (Tregs) in the tumour microenvironment. Stool transfer from responders to germ-free mice results in better tumour regression than that from non-responders. Taxonomical and functional differences have also been observed among patients who develop immunotherapy-related toxicity. Higher abundance of Firmicutes and low abundance of Bacteroidetes was associated with an increased risk of checkpoint blockade-induced colitis, thought to be mediated by low levels of immunosuppressive Tregs [66, 67].

## Obesity and the immune response

Epidemiological studies demonstrate a robust link between obesity and CRC development [68, 69]. Obesity-related carcinogenesis has been attributed to aberrant metabolic and immunological activity [70]. Furthermore, obesity is associated with immune dysfunction. Adipose tissue harbours a unique collection of innate lymphoid cells consisting of natural killer (NK) cells and a population of non-MHC restricted ‘unconventional’ T cells including invariant NKT (iNKT) cells, yδ T cells and mucosal-associated invariant T (MAIT) cells, that are key to maintaining immune homeostasis [71, 72]. In obesity, elevated levels of cytokines (IL-6, TNF and IL-1ß) transform the normally homeostatic anti-inflammatory environment into one that is pro-inflammatory and pathogenic. Overproduction of hormones (e.g. oestrogen), adipokines (e.g. leptin), and insulin that promote tumour cell survival and proliferation leads to tumour growth [73]. Adipose resident immune cells are depleted and metabolic re-programming impairs their anti-tumour function [74, 75]. Following bariatric surgery, the risk of CRC among individuals with obesity may be reduced to that of the general population [76, 77].

## Oncotherapeutic synergism

**Fig. 2:**
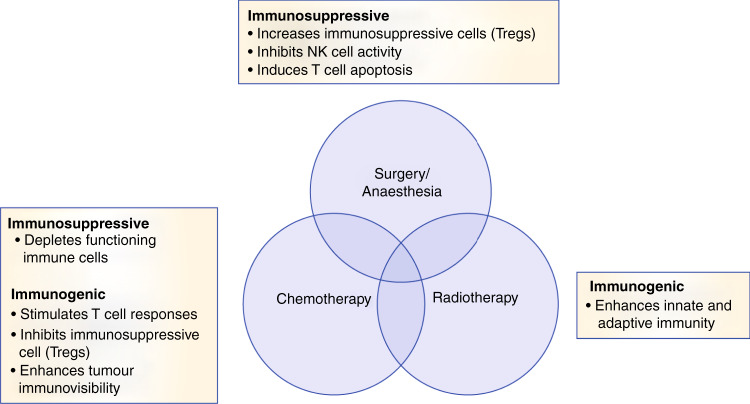
The immunomodulatory effects of surgery, chemotherapy and radiotherapy. The various aspects of cancer treament have both immunogenic and immunosuppressive properties.

Modern oncotherapeutics focused on exploiting the potential synergy of conventional (chemotherapy, radiotherapy) and novel (monoclonal antibodies) treatments to enhance tumour immunogenicity. In-depth understanding of the effects of each therapy on the immune response is essential when designing combined modality strategies (Fig. 2). Cytotoxic chemotherapy is one apex of the traditional tripartite of CRC treatment, alongside radiotherapy and surgery, that exerts a myriad of immunomodulatory effects. Although historically considered as immune depleting, cytotoxic chemotherapy activates anti-tumour immune responses by directly stimulating T-cell responses, inhibiting immunosuppressive cells (Tregs and myeloid-derived suppressor cells), and enhancing tumour immunovisibility [78, 79]. Pre-clinical and clinical studies found immunogenic cell death with several chemotherapeutic agents [80, 81]. In fact, recent data suggests intra-tumoural immune response as measured by the Immunoscore may predict the therapeutic benefit of adjuvant oxaliplatin-based chemotherapy in patients with stage III colon cancer [82].

Radiotherapy (RT) remains an integral component of treatment for locally advanced rectal cancer (LARC) as it reduces the risk of local recurrence when given preoperatively [3]. In addition to exerting direct genotoxic effects on tumour cell DNA (culminating in apoptosis), RT enhances innate and adaptive immune signalling pathways in the local tumour environment. Furthermore, it stimulates a systemic immunogenic response through circulating chemokines and cytokines [83], which may account for the regression of distant metastases outside the irradiation field, a phenomenon termed the abscopal effect [84].

## Neoadjuvant therapy

Colon and rectal cancers, and right and left sided colon cancers, differ in terms of molecular landscape, response to treatment and disease recurrence patterns [85, 86]. These differences are reflected in the different management approaches of each. Whilst colon cancer is generally treated with upfront surgery followed by adjuvant chemotherapy for stage III disease and select cases of stage II disease, rectal cancer requires more complex treatment consisting of neoadjuvant therapy, interval surgery and adjuvant chemotherapy. Neoadjuvant chemoradiotherapy is the standard of care for LARC (defined as bulky cT3/4 tumours or predicted node positive disease). It facilitates tumour downstaging, improves resectability and reduces local recurrence. Traditionally, 5 fluorouracil-based chemotherapy has been administered as a radiosensitising agent. This combination enhances local immune responses evidenced by a higher density of cytotoxic TILs that may be associated with better disease-free survival [87–89]. Individual response to nCRT however varies, with up to 25% of patients achieving a pCR, defined as absence of viable tumour cells in the resected specimen (ypT0N0) [90]. Immunological factors associated with pathological response remain poorly understood, and involve a complex interplay between various T-cell subsets and the tumour microenvironment. High pre-treatment CD8 + T-cell density, high CD4 + T-cell density and low Myeloid-Derived Suppressor Cell density is associated with a higher likelihood of tumour regression and achieving a pCR [87, 91, 92].

Total neoadjuvant therapy, whereby some or all of the planned chemotherapy (typically oxaliplatin) is given as either an induction or consolidation strategy, represents a promising option for LARC [93]. Two phase III trials presented showed that neoadjuvant chemotherapy is effective is this setting [94, 95]. While favourable short-term outcomes include improved chemotherapy compliance and superior pathological response, the impact on long-term disease-specific outcomes remains to be defined (although preliminary results suggest a survival benefit) [96–98]. Data on the immune effect of neoadjuvant chemotherapy is limited. A small pilot study found that FOLFOX was associated with increases in T-cell infiltration, and MHC-I and PD-1 expression compared to pre-treatment levels, suggesting CT-mediated priming of the tumour-immune microenvironment [99]. No correlation with pathological response was observed.

## The impact of multimodal therapy on anti-tumour immune response

How altered immune responses recover following systemic chemotherapy, either in the neoadjuvant or adjuvant setting, remains poorly understood and is not considered in current treatment strategies. Depletion of all the main subtypes of circulating lymphocytes has been observed for up to 12 months following standard chemotherapy in non-colorectal cancers [100]. It would seem counterintuitive to induce immunosuppression at a time when the immune system should be at maximal functional capacity. Whilst chemotherapy activates anti-tumour immune responses, it may also deplete functional immune cells. Understanding the underpinning molecular mechanisms of treatment-related immune dysfunction may guide strategy approach (e.g. neoadjuvant vs adjuvant therapy).

Evaluation of the immune response to surgery has focused on mechanisms that drive post-operative recovery including single cell analysis for unique signatures that predict it [101]. Surgical-induced stress evokes an immunosuppressive environment stimulating the inflammatory cascade with secretion of chemokines and cytokines and increasing levels of Tregs [102]. Does this lead to a permissive environment where tumour cells could thrive? It is plausible because Treg proliferation hinders anti-tumour surveillance and perioperative NK cell cytotoxicity may be impaired for up to 2 months [103].

General anaesthesia may also evoke immune modulation independent of surgical insult. Both inhalational and intravenous anaesthetic agents inhibit NK cell activity, induce T-cell apoptosis, and enhance angiogenesis through hypoxia inducible factor-1α (HIF-1α) [104]. The magnitude of these effects varies depending on the specific agent used. There is evidence that intravenous anaesthesia induces less immunosuppression than inhalational and regional agents such that it may be a preferred approach for oncological surgery [105]. In addition, regional anaesthesia use should reduce perioperative opioid requirements to minimise transient opiate-induced suppression of NK cytotoxicity [105].

Perioperative allogenic blood transfusion is associated with an increased likelihood of disease recurrence and cancer-specific mortality in CRC [106, 107]. It has been postulated that the association between blood transfusion and cancer recurrence is due to transfusion-related immune modulation (TRIM). TRIM is a biological phenomenon characterised by induction of suppressor T cells, inhibition of NK cell function and polarisation of the immune system to Th2 response, with suppression of Th1 response [108]. Enhanced systemic inflammation (higher levels of IL-6) and decreased immunity (lower CD8 + T-cell counts) were observed post-transfusion in patients undergoing resection for CRC [109].

Despite undergoing surgery with curative intent, up to one third of patients with CRC will develop distant metastases [2]. It is possible that surgery and anaesthesia-mediated immunosuppression enhance tumour dissemination. The presence of circulating tumour cells (CTCs) in peripheral blood at least 24 h after resection of CRCs represents an independent prognostic marker of disease recurrence [110]. Curative surgery may paradoxically create an opportunity for CTCs to evade eradication by exploiting perioperative immune dysfunction. Discerning whether immunosuppression is mediated by surgical insult or anaesthesia is challenging. Simple measures include adoption of minimally-invasive surgery, use of select anaesthetic agents, and adherence to enhanced recovery pathways to reduce the adverse effects of perioperative immunomodulation for optimal cancer care.

## Conclusion

The therapeutic field of immuno-oncology is rapidly gaining momentum, with strikingly promising results observed in clinical practice. Re-programming the immune system to enhance anti-tumour response has revolutionised cancer therapy. CRC presents a major challenge for cancer immunotherapy. Dichotomisation into MSI and MSS provides limited prognostic and therapeutic information. Each group is immunologically heterogeneous. Additionally, overlap between the two groups exists in terms of immune infiltrate. In-depth understanding of tumour biology and immunology, utilising the synergy of chemotherapeutic agents and immunotherapies, and identifying prognostic and predictive immunological biomarkers will enable the delivery of precision and personalised cancer care. By considering each apex of the tripartite of cancer management (oncotherapeutics, radiotherapy and surgery), an era of unprecedented disease control, survivorship and cure rates is on the horizon.

## Data Availability

The data used in this review are available in PubMed, Embase and Scopus databases.
